# Quality Initiatives May Affect Diagnostic Accuracy: STEMI Mimics in an Age of Decreasing Door to Balloon Time

**DOI:** 10.5811/cpcem.2016.12.33009

**Published:** 2017-03-15

**Authors:** Jose L Villa-Uribe, Elizabeth M Schoenfeld

**Affiliations:** * University of Massachusetts Medical School-Baystate, Department of Emergency Medicine, Springfield, Massachusetts; † University of Massachusetts Medical School-Baystate, Department of Emergency Medicine, Center for Quality of Care Research, Springfield, Massachusetts

A 53-year-old male with several cardiac risk factors presented to the emergency department with back pain and an electrocardiogram concerning for an anterior ST-elevation myocardial infarction. The patient decompensated hemodynamically and a point-of-care ultrasound revealed a small pericardial effusion. An aortic dissection was ruled out by computed tomography angiography and coronary catheterization did not reveal a culprit lesion. The diagnosis of tamponade was made in the catheterization laboratory after measurement of intra-cardiac diastolic pressures and the patient’s symptoms resolved after drainage of 100 mL of pericardial fluid.

## INTRODUCTION

ST-segment elevation in an electrocardiogram (ECG) immediately suggests ST-elevation myocardial infarction (STEMI) as a likely diagnosis for patients with cardiac symptoms. This ECG finding, however, can be present in a variety of other entities such as myocarditis, pericarditis, vasospasm, aortic dissection, cardiac tamponade, Brugada syndrome, left bundle branch block, and intracranial hemorrhage.^1^–^8^ Providers need to consciously and consistently consider these alternative diagnoses as they face continued emphasis on improving door-to-balloon (DTB) times for patients with STEMIs. In this article, we report a case of cardiac tamponade that presented with ST-segment elevation.

## CASE REPORT

A 53-year-old male with a history of hypertension, hyperlipidemia, and type-2 diabetes mellitus presented to our emergency department (ED) with a three-day history of worsening back pain. The pain was diffuse in the thoracic and lumbar areas and did not improve despite ibuprofen and oxycodone/acetaminophen. The patient denied chest pain, shortness of breath, fevers, chills, or any traumatic injuries. He had been evaluated at an urgent care center twice since the onset of his symptoms.

**Image 1:**
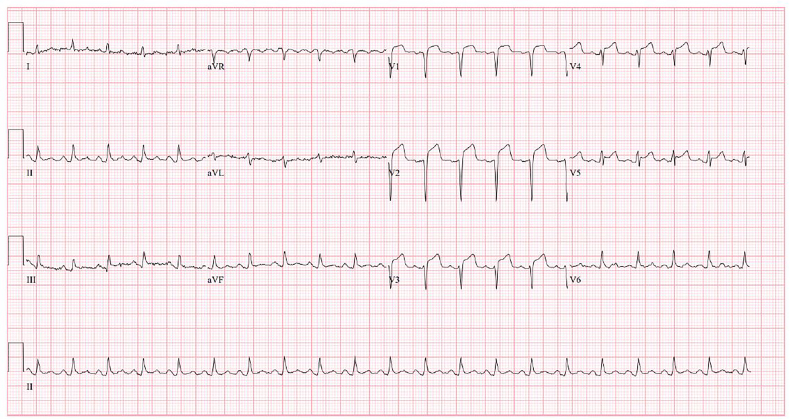
Electrocardiogram showed ST-segment elevation in leads V1–V5

**Image 2:**
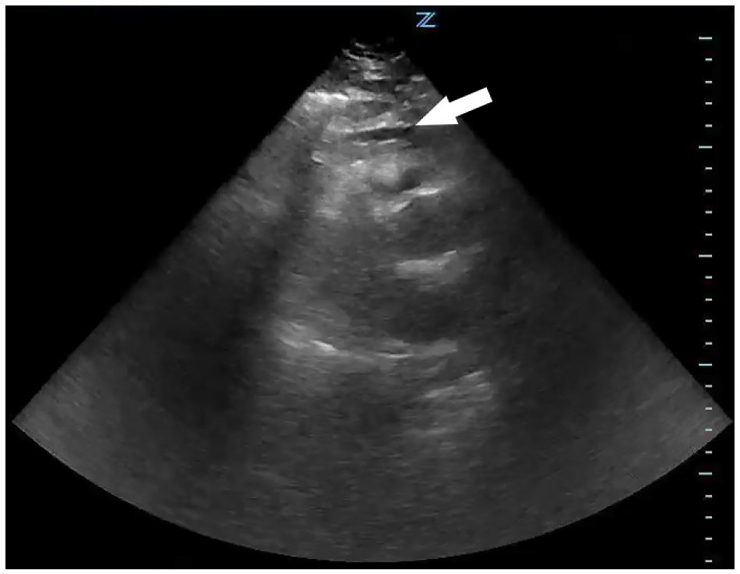
Point-of-care cardiac ultrasound, parasternal long axis view, showing a small pericardial effusion (arrow). See the supplemental file “Video1.mp4” for a video of this ultrasound.

On arrival to the ED, the patient was afebrile, had a blood pressure of 104/69 mmHg, and a heart rate of 125 bpm. An ECG revealed ST-segment elevation in leads V1–V5 (Image 1) and the cardiac catheterization laboratory was activated due to concern for an anterior STEMI. During this time, the patient’s blood pressure decreased to 85/74 mmHg, which did not increase despite a one-liter bolus of normal saline. A bedside cardiac echocardiogram performed by the ED provider showed a small pericardial effusion (Image 2) and a portable upright chest radiograph showed a widened mediastinum (11 cm). The patient’s hypotension and imaging findings were concerning for an aortic dissection, leading the ED provider, in conjunction with the interventional cardiologist, to obtain an emergent computed tomography angiography (CTA) of the chest. The patient was started on peripheral norepinephrine given his continued hemodynamic instability and, after the CTA was negative for dissection, he was transported to the catheterization laboratory.

**Image 3:**
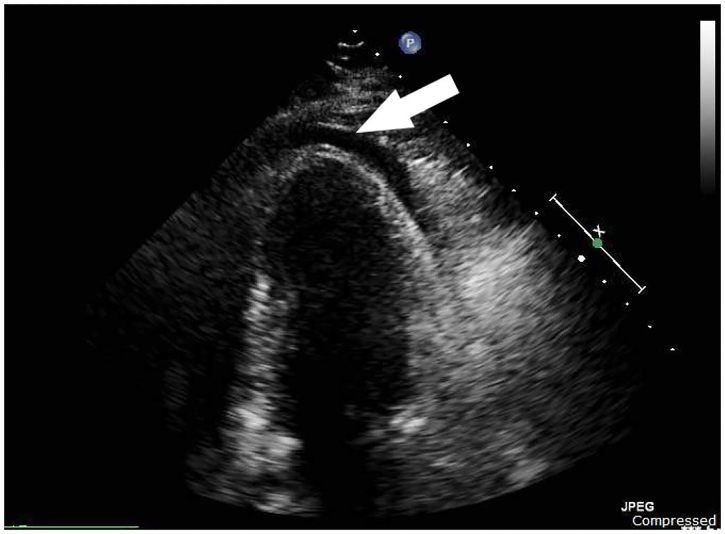
Emergent transthoracic echocardiogram performed in the cardiac catheterization lab prior to pericardiocentesis: two-chamber apical view showing a small pericardial effusion (arrow). See the supplemental file “Video2.mp4” for a video of this ultrasound.

An intra-aortic balloon pump was placed in the catheterization lab, and evaluation of the coronary arteries showed minimal non-obstructive coronary disease. An emergent (cardiologist-performed) transthoracic echocardiogram in the catheterization lab once again showed a small circumferential pericardial effusion (Image 3). A Swan-Ganz catheter was placed and showed equalization of diastolic pressures across the cardiac chambers. A pericardiocentesis was then performed with drainage of 100 mL of straw-colored fluid, after which the patient’s mean arterial pressure improved to 95 mmHg off vasopressors. This procedure also led to immediate relief of his back pain. A pericardial drain was left in place, the intra-aortic balloon pump was removed, and the patient was transported to the cardiac intensive care unit for further management.

Lab work revealed an elevated troponin T (0.15 ng/mL) with normal creatinine kinase (36 U/L), elevated white blood cell count (19,900/mm^3^), elevated erythrocyte sedimentation rate (84 mm/hr), and elevated C-reactive protein (39.1 mg/dL), suggesting the diagnosis of pericarditis. The patient was started on colchicine and ibuprofen and continued to be hemodynamically stable through his hospital stay. No infectious, malignant, traumatic, pharmaceutical, or autoimmune factors were identified as the etiology for his pericarditis during his hospital stay. A repeat echocardiogram showed resolution of the pericardial effusion. The pericardial drain was removed and the patient was discharged on hospital day three with scheduled outpatient follow up.

## DISCUSSION

We present a case of pericardial tamponade presenting as a possible STEMI. Although this patient’s symptoms were atypical and the ECG did not show reciprocal changes, his comorbidities and ST-elevations made acute coronary syndrome (ACS) the leading concern. The clinicians retained their skepticism and identified aortic dissection as an alternative diagnosis as his clinical picture evolved. Once this was ruled out, however, the focus immediately returned to ACS. In the end, the final diagnosis of tamponade was not made without invasive testing. This case highlights the need for providers to remain vigilant for alternative diagnoses in patients with presumed STEMIs despite the pressures to decrease DTB time.

Lower DTB times are known to improve mortality in STEMI,^9^–^11^ and many initiatives around the world have successfully decreased these times.^12^–^14^ These aggressive efforts to improve DTB time, however, can predispose healthcare providers to cognitive errors such as anchoring or premature closure when an ECG shows ST-segment elevations in a patient with a plausible history of present illness. A recent study by Fanari et. al. quantifies this effect: while an aggressive DTB improvement initiative did lead to lower times to therapy (76 to 61 minutes, p=0.001), the rate of false-positive STEMI (FP-STEMI) increased significantly (7.7% to 16.5%, p=0.02). More concerning was the increase of in-hospital mortality for FP-STEMI (5.6% to 21.6%, p=0.03). These preliminary findings led to a quality improvement initiative aimed at recognizing non-STEMI causes of ST-segment elevation along with the pre-existing DTB initiative. Although the rate of FP-STEMI remained high after implementation (16.5% to 20.3%, p=0.30), there was a significant decrease in hospital mortality for this group (21.6% to 4.5%, p=0.03).^15^ These results are encouraging in showing that decreasing FP-STEMI mortality is possible in the era of ever-decreasing DTB times.

##
